# Perceived ethnic discrimination, suicidal ideation and mastery in a multi-ethnic cohort: the HELIUS study

**DOI:** 10.1192/bjo.2022.640

**Published:** 2023-01-20

**Authors:** Fabienne E. M. Willemen, Caroline B. B. C. M. Heuschen, Jasper B. Zantvoord, Henrike Galenkamp, Matty A. S. de Wit, Aeilko H. Zwinderman, Damiaan A. J. P. Denys, Claudi L. H. Bockting, Karien Stronks, Anja Lok

**Affiliations:** Department of Psychiatry, University of Amsterdam, Amsterdam University Medical Centres (UMC), Amsterdam, The Netherlands; Department of Psychiatry, University of Amsterdam, Amsterdam University Medical Centres (UMC), Amsterdam, The Netherlands; and Department of Child and Adolescent Psychiatry, Amsterdam Neuroscience, University of Amsterdam, Amsterdam University Medical Centres (UMC), Amsterdam, The Netherlands; Department of Public and Occupational Health, University of Amsterdam, Amsterdam University Medical Centres (UMC), Amsterdam, The Netherlands; Department of Epidemiology, Health Promotion and Care Innovation, Public Health Service Amsterdam, Amsterdam, The Netherlands; Center for Urban Mental health, University of Amsterdam, Amsterdam, The Netherlands; Department of Psychiatry, University of Amsterdam, Amsterdam University Medical Centres (UMC), Amsterdam, The Netherlands; and Center for Urban Mental health, University of Amsterdam, Amsterdam, The Netherlands; Department of Public and Occupational Health, University of Amsterdam, Amsterdam University Medical Centres (UMC), Amsterdam, The Netherlands; and Center for Urban Mental health, University of Amsterdam, Amsterdam, The Netherlands

**Keywords:** HELIUS study, perceived ethnic discrimination, suicidal ideation, ethnicity, mastery

## Abstract

**Background:**

The association between perceived ethnic discrimination (PED) and mental health conditions is well studied. However, less is known about the association between PED and suicidal ideation, or the role of positive psychosocial factors in this association.

**Aims:**

To examine the association between PED and suicidal ideation among ethnic minority groups in Amsterdam, The Netherlands, and investigate whether ethnicity and mastery (people's extent of feeling in control of their lives and environment) moderate this association.

**Method:**

Cross-sectional data from the multi-ethnic HELIUS study were analysed (*n* = 17 053) for participants of South-Asian Surinamese, African Surinamese, Ghanaian, Turkish and Moroccan origin. PED was measured using the Everyday Discrimination Scale, suicidal ideation using item 9 of the Patient Health Questionnaire-9 and mastery using the Pearlin–Schooler Mastery Scale.

**Results:**

Logistic regression analyses demonstrated a small positive association between PED and suicidal ideation (OR = 1.068, 95% CI 1.059–1.077), which did not differ among ethnic minority groups. Mastery did not moderate the association between PED and suicidal ideation among the ethnic minority groups.

**Conclusions:**

Our findings support the hypothesis that PED is associated with suicidal ideation and this association does not significantly vary between ethnic minority groups. Although higher levels of mastery were associated with lower suicidal ideation, mastery did not moderate the relationship between PED and suicidal ideation. Besides targeting ethnic discrimination as a societal problem, future longitudinal research is needed to investigate whether interventions aimed at improving mastery could reduce suicidal ideation in ethnic minority groups.

Suicidality is a major global health challenge, with more than 700 000 individuals dying by suicide each year.^1^ Suicidal ideation and behaviour are mostly associated with mental disorders, in particular major depressive disorder.^2^ However, previous insights suggest that mental disorders are not the sole risk factor determining suicidality and that suicidal ideation and behaviour rather depend on many interacting sociodemographic and psychosocial factors.^3,4^ Furthermore, rates of suicidal ideation and behaviour vary within and between geographical regions, across cultures and ethnic (minority) groups.^5^ Together, this emphasises the complex and multifactorial aetiology of suicidal ideation and behaviour. Growing evidence suggests the influence of perceived ethnic discrimination (PED) on suicidal ideation.^6,7^ PED is seen as a chronic stressor that represents an individual's day-to-day subjective perception of experiences of overt and subtle acts of unfair treatment because of their ethnic background.^8^ Ethnic discrimination is widely prevalent in the European Union (EU) and the USA, and thus constitutes a major social problem.^9,10^ Early studies showed an association between PED and negative mental health outcomes that differed between ethnic groups.^11,12^ However, the association between PED and suicidal ideation is less well studied and only investigated in the USA.^13–16^ The populations studied in the USA are not comparable to those in other geographical regions because of the differences in ethnic minority groups living in different regions and their position in society.^11,13^

Furthermore, a previous study showed that positive psychosocial factors, such as mastery, might have a protective effect on suicidal ideation.^17^ Mastery is the extent to which people feel in control of their lives and environment and are able to bring about desired outcomes.^17^ The study investigated the moderating effect of mastery on the association between PED and depression, which was robustly present across ethnic minority groups in The Netherlands.^17^ However, the effect of mastery on the association between PED and suicidal ideation has not yet been studied. Previous studies have shown that levels of mastery may differ between ethnic groups. The study in The Netherlands^17^ found, for instance, different levels of mastery between Surinamese, Turkish, Moroccan and Ghanaian ethnic groups, which might be caused by different interpretations of control among ethnic minority groups.^18^ Another study showed that self-control interventions can improve mastery among ethnic minority groups.^19^ If mastery moderates the relationship between PED and suicidal ideation, this could in turn lead to different results between ethnic minority groups. Therefore, further research in Europe on the association between PED and suicidal ideation among ethnic minorities and the role of mastery is warranted. This might generate innovative ideas on how to reduce suicidal ideation from a societal perspective, by targeting discrimination, and from an individual perspective, by improving mastery. Hence, we explored the association between PED, suicidal ideation and the potential moderating role of ethnicity and mastery among a multi-ethnic cohort in The Netherlands. More specifically, we hypothesised that: (a) an association exists between PED and suicidal ideation among ethnic minority groups in The Netherlands; (b) the association between PED and suicidal ideation differs among ethnic minority groups; and (c) high levels of mastery weaken the association between PED and suicidal ideation among ethnic minority groups.

## Method

### Study design

We used data from the Healthy Life in an Urban Setting (HELIUS) study, which is a multi-ethnic cohort study conducted in Amsterdam, The Netherlands. A description of the full study protocol can be found elsewhere.^20,21^ In the HELIUS study, baseline data are available for men and women in the age range of 18 to 70 years from six different ethnic backgrounds, namely Dutch, South-Asian Surinamese, African Surinamese, Moroccan, Turkish and Ghanaian; these are largest ethnic groups in Amsterdam. Through the municipality registry of Amsterdam, random sampling, stratified by ethnic origin, was performed.

### Study population

The HELIUS study includes 24 789 participants in total, who were enrolled between January 2011 and December 2015. Data were obtained using an extensive questionnaire and a physical examination. Participants were eligible for the current study if they were non-Dutch (since the focus in this paper is on PED) and had completed the Everyday Discrimination Scale (EDS),^22^ item 9 (suicidal ideation) of the Patient Health Questionnaire-9 (PHQ-9)^23^ and the Pearlin–Schooler Mastery Scale (PMS).^24^ A total of 17 053 participants (3043 South-Asian Surinamese, 4151 African Surinamese, 2339 Ghanaian, 3614 Turkish and 3906 Moroccan) met these criteria. Prior to data collection, written consent was obtained from all participants. The authors assert that all procedures contributing to this work comply with the ethical standards of the relevant national and institutional committees on human experimentation and with the Helsinki Declaration of 1975, as revised in 2008. All procedures involving human subjects/patients were approved by the Institutional Ethical Board of the Amsterdam UMC, location AMC (MREC 10/100# 17.10.1729).

### Measurements

#### Assessment of perceived ethnic discrimination

PED was measured as the perception of daily discriminatory experiences using the validated EDS.^22^ The EDS consists of nine items assessed on a 5-point Likert scale (1 ‘never’ to 5 ‘very often’). The Cronbach's alpha was 0.88.^22^ The questionnaire was adapted to individuals’ experience of discrimination due to their ethnic background, as described earlier in the HELIUS study.^25^ We created sum scores based on the response on all nine items (theoretical range 9–45). If one item was missing, we first calculated a mean score based on available items and converted this to a sum score by multiplying it by nine. If more than one item was missing, the sum score was coded as missing.^25^

#### Assessment of suicidal ideation

Suicidal ideation was assessed using item 9 of the PHQ-9 (‘Over the past two weeks, how often have you been bothered by thoughts that you would be better off dead or of hurting yourself in some way?’). Responses could be given on a 4-point Likert scale from 1 (not at all) to 4 (nearly every day). The 4-point Likert scale was transformed into categories indicating absence of suicidal ideation (item 9 score of 1) and presence of suicidal ideation (item 9 score >1, i.e. more than a few days). The PHQ-9 has been validated for all ethnic groups included in the HELIUS study and had a Cronbach's alpha of 0.89.^23,26^ The use of this single item for assessing suicidal ideation was validated in a study by Walker et al (2010).^27^

#### Assessment of mastery

Mastery was measured using the abbreviated version of the validated PMS, which has a Raykov's reliability coefficient (rho) of 0.90 and is commonly used to measure levels of mastery.^24,28^ Only the five negatively phrased items were utilised, with response categories on a 5-point Likert scale (1 ‘totally disagree’ to 5 ‘totally agree’) (e.g. ‘I have little control over the things that happen to me’). A sum score of the five items was used in the analyses (theoretical range 5–25). Missing values were managed using the method described above.^25^

### Covariates

#### Sociodemographic factors

Based on the literature, various sociodemographic variables which have been associated with suicidal ideation were included as potential confounding variables.^3,29–32^ These were age, gender, ethnicity, socioeconomic status and marital status. Ethnicity was defined by the country of birth of participants and of their parents.^33^ More specifically, participants were defined as of non-Dutch ethnic origin if they fulfilled one of two criteria: (a) they were born outside The Netherlands and had at least one parent born outside The Netherlands (first generation) or (b) they were born in The Netherlands but both parents were born outside The Netherlands (second generation). After data collection, participants of Surinamese ethnic origin were further classified according to self-reported ethnic origin (obtained by questionnaire) into ‘African’, ‘South-Asian’ or ‘other’.^21^ Two factors were used as indicators of socioeconomic status, namely the highest level of education completed and the current employment status. Educational level was divided into four categories: no education or elementary education; lower vocational and general secondary education; intermediate vocational and higher secondary education; and higher vocational and university education. Employment status was categorised into the following categories: employed; not in the labour force; unemployed; incapacitated. Marital status was assessed as: married/living together; divorced/separated/widowed; or never married.

#### Other covariates

We included religiosity, lifestyle factors and antidepressant use to adjust for confounding in all analyses. First, religiosity (‘Do you practise a specific religion right now?’: yes/no) was added. Second, lifestyle factors, including smoking, alcohol dependence and cannabis dependence, were all individually added as confounders. Last, antidepressant use (modern antidepressants, mood stabilisers or tricyclic antidepressants: yes/no) was added, simultaneously with lifestyle factors. Antidepressant use was positive if the participant used one or more antidepressant.

### Data analysis

**Fig. 1:**
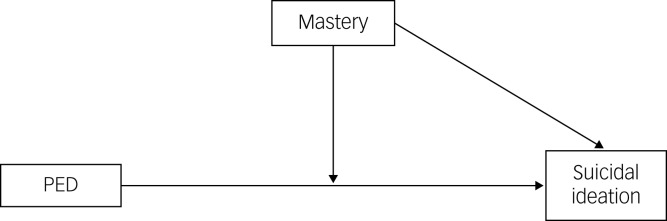
The proposed pathway of the main association between perceived ethnic discrimination (PED) and suicidal ideation, showing the moderating role of mastery.

First, preliminary analyses were performed, including evaluating missing data, calculating descriptive statistics separately for the five ethnic groups and calculating correlations between variables. Missing values not handled as described above were deleted using listwise deletion, because less than 2% were missing for each variable. Fig. 1 shows the proposed pathways of our main variables, namely suicidal ideation, PED and mastery. All analyses were carried out with SPSS version 26.0 for Windows.

#### Hypothesis testing

For the first hypothesis (an association exists between PED and suicidal ideation among ethnic minority groups in The Netherlands) we determined the (cross-sectional) association between PED and suicidal ideation using logistic regression analysis.

For the second hypothesis (the association between PED and suicidal ideation differs among ethnic minority groups) we examined differences in the association between PED and suicidal ideation in the five ethnic minority groups by performing logistic regression analysis including an interaction of PED and ethnicity (ethnicity × PED) (four degrees of freedom test). Subsequently, stratification by ethnicity was performed to show the variations in the individual estimates for the five ethnic minority groups.

For the third hypothesis (high levels of mastery weaken the association between PED and suicidal ideation among ethnic minority groups) a three-way interaction of level of mastery, PED and ethnic minority group was done to investigate whether this relationship differs across ethnic minority groups. If the three-way interaction was significant, a logistic regression analysis was performed including an interaction of PED × mastery (sum score) separately for each of the five ethnic minority groups. If this interaction was significant, the influence of mastery was examined by stratifying for high and low levels of mastery (using a median split) within ethnic minority groups. If the mastery × PED × ethnicity interaction was non-significant, we removed this term and used the remaining more parsimonious models (mastery × PED).

In all analyses, the abovementioned confounders were hierarchically added and three primary models were used in total. In model 1, age and gender were added to the regression analyses. Model 2 was additionally corrected for ethnicity, religiosity, socioeconomic status and marital status. In model 3, additional adjustments were made for lifestyle factors and antidepressant use. The results shown here are based on model 3 since this model accounts for most potential confounding effects and lifestyle factors. Where crude analyses for all hypotheses were carried out, we also show the crude model in the results (the crude model ignores potential confounders).

#### Sensitivity analyses

We conducted separate sensitivity analyses by adding depressive symptoms as a confounder to all analyses. ‘Depressive symptoms’ were measured using the first eight items of the PHQ-9 and calculated as the sum score.^23^ Missing values for depressive symptoms were handled as described above.^25^

## Results

In total, 17 053 participants were included in the analyses; 41% were male and the mean age for the total cohort was 44 years (range 18–70 years). The PED rates were similar across the five ethnic groups, with a sum score mean of 18.8 (s.d. = 4.4, range 9–45). Presence of suicidal ideation differed substantially across the ethnic groups and was lowest in the groups of African Surinamese and Ghanaian ethnic background. Mean scores for mastery were roughly similar among the ethnic minority groups. Table 1 shows baseline characteristics of the study population stratified by ethnicity.

**Table 1:** Baseline characteristics of participants by ethnicity

	South-Asian Surinamese (*n* = 3043)	African Surinamese (*n* = 4151)	Ghanaian (*n* = 2339)	Turkish (*n* = 3614)	Moroccan (*n* = 3906)
Suicidal ideation, yes: *n* (%)	375 (12.3)	261 (6.3)	110 (4.7)	348 (9.6)	323 (8.3)
Missing	18 (0.6)	41 (1.0)	51 (2.2)	46 (1.3)	53 (1.4)
Perceived ethnic discrimination, mean (s.d.)^a^	17.8 (6.8)	18.4 (6.8)	16.9 (7.1)	16.7 (6.6)	17.9 (7.1)
Missing (%)	22 (0.7)	41 (1.0)	42 (1.8)	52 (1.4)	38 (1.0)
Mastery, mean (s.d.)^b^	18.4 (4.3)	19.5 (4.0)	19.4 (4.0)	18.3 (5.2)	18.3 (4.4)
Missing (%)	17 (0.6)	37 (0.9)	44 (1.9)	43 (1.2)	37 (0.9)
Male, *n* (%)	1371 (45.1)	1616 (38.9)	905 (38.7)	1634 (45.2)	1514 (38.8)
Age, years: mean (s.d.)	45.5 (13.4)	47.9 (12.5)	44.7 (11.2)	40.4 (12.2)	40.5 (12.9)
Marital status, *n* (%)					
Married/living with a partner	1354 (44.5)	1207 (29.1)	847 (36.2)	2340 (64.7)	2395 (61.3)
Divorced/separated/widowed	672 (22.1)	682 (16.4)	679 (29.0)	497 (13.8)	483 (12.4)
Never married	1001 (32.9)	2231 (53.7)	779 (33.3)	761 (21.1)	1010 (25.9)
Missing	16 (0.5)	31 (0.7)	34 (1.5)	16 (0.4)	18 (0.5)
Educational level, *n* (%)^c^					
Low	437 (14.4)	231 (5.6)	660 (28.2)	1135 (31.4)	1205 (30.8)
Medium-low	1010 (33.2)	1477 (35.6)	917 (39.2)	889 (24.6)	694 (17.8)
Medium-high	885 (29.1)	1464 (35.3)	578 (24.7)	1020 (28.2)	1294 (33.1)
High	695 (22.8)	943 (22.7)	142 (6.1)	532 (14.7)	675 (17.3)
Missing	16 (0.5)	36 (0.9)	42 (1.8)	38 (1.1)	38 (1.0)
Employ status, *n* (%)					
Employed	1858 (61.1)	2598 (62.6)	1369 (58.5)	1871 (51.8)	1895 (48.5)
Not in the labour force	431 (14.2)	486 (11.7)	157 (6.7)	823 (22.8)	1057 (27.1)
Unemployed	442 (14.5)	648 (15.6)	557 (23.8)	536 (14.8)	604 (15.5)
Incapacitated	279 (9.2)	377 (9.1)	203 (8.7)	317 (8.8)	311 (8.0)
Missing	33 (1.1)	42 (1.0)	53 (2.3)	67 (1.9)	39 (1.0)
Religiosity, *n* (%)					
Currently practising	2428 (79.8)	3191 (76.9)	1992 (85.2)	3362 (92.0)	3811 (97.6)
Missing	18 (0.6)	25 (0.6)	91 (3.9)	36 (1.0)	18 (0.5)
Antidepressant use, yes: *n* (%)					
Missing	113 (3.7)	98 (2.4)	35 (1.5)	202 (5.6)	156 (4.0)
		1 (0.0)		1 (0.0)	4 (0.1)
Depressive symptoms, mean (s.d.)^d^					
Missing (%)	0.65 (0.69)	0.48 (0.55)	0.41 (0.52)	0.78 (0.72)	0.72 (0.68)
	20 (0.7)	38 (0.9)	53 (2.3)	48 (1.3)	43 (1.1)

a. Range 9 (lowest) to 45 (highest).

b. Higher scores indicate lower degrees of mastery.

c. Low, no education or elementary education; medium-low, lower vocational and general secondary education; medium-high, intermediate vocational and higher secondary education; high, higher vocational education or university.

d. Higher scores indicate more depressive symptoms.

Table 2 reports the zero-order correlations of the main variables, namely suicidal ideation, PED and mastery. Significant moderate correlations were identified among the main variables. PED was positively correlated with suicidal ideation but negatively correlated with mastery. Mastery was negatively correlated with suicidal ideation. All analyses model the likelihood of suicidal ideation as a function of PED and other factors. The results of hypotheses one and two are reported for model 3, in which adjustments were made for all confounders. In model 4, the results are presented for the sensitivity analyses with depressive symptoms as confounder.

**Table 2:** Zero-order correlations of suicidal ideation, perceived ethnic discrimination and mastery

Variable	1	2	3
1 Suicidal ideation	−		
2 Perceived ethnic discrimination	0.522**	−	
3 Mastery	−0.351**	−0.554**	−

** *P* < 0.01 (two-sided).

### The association between PED and suicidal ideation among ethnic minority groups in The Netherlands

Table 3 shows the association between PED and suicidal ideation. PED was positively associated with suicidal ideation (OR = 1.068, 95% CI 1.059–1.077). After adjustment for confounders (model 1: adjusted for age and gender; model 2: adjusted for ethnicity, religiosity, socioeconomic status and marital status; model 3: adjusted for lifestyle factors and antidepressant use), only small changes appeared in the odds ratios (Table 3). The explained variance of the fully adjusted model (model 3) assessed using Nagelkerke's *R*^2^ was 14%.

**Table 3:** Suicidal ideation as a function of perceived ethnic discrimination^a^

	OR	*P*	95% CI	
			Lower	Upper
Crude model	1.068	<0.001	1.060	1.076
Model 1	1.070	<0.001	1.062	1.078
Model 2	1.070	<0.001	1.062	1.079
Model 3	1.068	<0.001	1.059	1.077
Model 4	1.033	<0.001	1.024	1.043

a. Model 1, adjusted for age and gender; Model 2, adjusted for ethnicity, religiosity, socioeconomic status and marital status; Model 3, adjusted for lifestyle factors and antidepressant use; Model 4, adjusted for depressive symptoms.

### The association between PED and suicidal ideation does not differ among ethnic minority groups

Overall, the interaction between the ethnic minority groups and PED was not significant (*P* = 0.067) (Table 4). After stratification by ethnicity, the analyses for each ethnic minority group were all statistically significant (Table 4). The explained variance (Nagelkerke's *R*^2^) varied between 11.4 and 19.3% in the fully adjusted model (model 3).

**Table 4:** Suicidal ideation as a function of perceived ethnic discrimination (PED), ethnicity^a^ and their interaction, and suicidal ideation as a function of PED by ethnic group^b^

	Crude model				Model 1				Model 2				Model 3				Model 4			
			95% CI				95% CI				95% CI				95% CI				95% CI	
	OR	*P*	Lower	Upper	OR	*P*	Lower	Upper	OR	*P*	Lower	Upper	OR	*P*	Lower	Upper	OR	*P*	Lower	Upper
PED	1.063	<0.001	1.047	1.080	1.065	<0.001	1.048	1.082	1.060	<0.001	1.043	1.077	1.058	<0.001	1.041	1.076	1.016	0.114	0.996	1.035
African Surinamese	0.300	<0.001	0.177	0.509	0.294	<0.001	0.173	0.499	0.306	<0.001	0.179	0.521	0.330	<0.001	0.193	0.566	0.355	<0.001	0.193	0.653
Ghanaian	0.169	<0.001	0.085	0.337	0.164	<0.001	0.082	0.327	0.113	<0.001	0.056	0.228	0.130	<0.001	0.064	0.263	0.196	<0.001	0.088	0.439
Turkish	0.830	0.431	0.521	1.321	0.796	0.337	0.499	1.269	0.775	0.295	0.481	1.249	0.737	0.217	0.454	1.196	0.427	0.003	0.245	0.743
Moroccan	0.628	0.058	0.388	1.016	0.594	0.034	0.366	0.962	0.552	0.019	0.337	0.905	0.598	0.044	0.363	0.986	0.346	<0.001	0.195	0.661
PED × ethnicity (d.f. = 4)		0.096				0.098				0.050				0.067				0.057		
PED × African Surinamese	1.019	0.116	0.995	1.044	1.020	0.105	0.996	1.045	1.109	0.129	0.995	1.044	1.015	0.227	0.991	1.040	1.031	0.033	1.002	1.061
PED × Ghanaian	1.033	0.037	1.002	1.065	1.034	0.033	1.003	1.065	1.044	0.007	1.012	1.076	1.044	0.006	1.012	1.077	1.048	0.010	1.011	1.087
PED × Turkish	0.999	0.960	0.977	1.022	1.001	0.954	0.978	1.023	1.003	0.879	0.979	1.025	1.003	0.801	0.980	1.027	1.009	0.518	0.982	1.037
PED × Moroccan	0.999	0.959	0.977	1.022	1.000	0.970	0.978	1.023	1.006	0.612	0.983	1.029	1.005	0.654	0.982	1.029	1.020	0.154	0.993	1.047
Associations between PED and suicidal ideation by ethnic group																				
South-Asian Surinamese	1.064	<0.001	1.047	1.081	1.066	<0.001	1.049	1.083	1.061	<0.001	1.044	1.079	1.060	<0.001	1.043	1.078	1.019	0.065	0.99	1.039
African Surinamese	1.084	<0.001	1.065	1.104	1.089	<0.001	1.069	1.109	1.084	<0.001	1.065	1.104	1.079	<0.001	1.059	1.099	1.051	<0.001	1.028	1.074
Ghanaian	1.099	<0.001	1.071	1.127	1.109	<0.001	1.080	1.139	1.113	<0.001	1.084	1.144	1.117	<0.001	1.086	1.144	1.076	<0.001	1.039	1.114
Turkish	1.062	<0.001	1.046	1.079	1.064	<0.001	1.047	1.082	1.060	<0.001	1.043	1.078	1.058	<0.001	1.041	1.076	1.024	0.014	1.005	1.043
Moroccan	1.063	<0.001	1.047	1.080	1.062	<0.001	1.045	1.079	1.063	<0.001	1.046	1.081	1.060	<0.001	1.042	1.079	1.029	0.004	1.009	1.050

a. Reference group: South-Asian Surinamese.

b. Model 1, adjusted for age and gender; Model 2, adjusted for ethnicity, religiosity, socioeconomic status and marital status; Model 3, adjusted for lifestyle factors and antidepressant use; Model 4, adjusted for depressive symptoms.

### Mastery does not moderate the association between PED and suicidal ideation among ethnic minority groups

The three-way interaction of level of mastery, PED and ethnic minority group was non-significant (*P* = 0.089), indicating that a potential moderating effect of mastery on the association between PED and suicidal ideation did not differ between the ethnic minority groups. In the subsequent parsimonious model the interaction between mastery and PED was also non-significant (OR = 1.001, 95% CI 0.999–1.003), indicating that mastery did not moderate the association between PED and suicidal ideation. The main effect of mastery on suicidal ideation remains significant (OR = 0.784, 95% CI 0.772–0.796), indicating that mastery has a protective effect on suicidal ideation.

### Sensitivity analyses

After depressive symptoms were added as a confounder (model 4 in all analyses), results regarding the first and second hypotheses were attenuated compared with the main analyses (Tables 3 and 4). Overall, the association between PED and suicidal ideation became less strong after adding depressive symptoms (Table 3 and 4).

## Discussion

This study found a positive, although small, association between PED and suicidal ideation which did not significantly differ across ethnic minority groups. In addition, mastery did not moderate the association between PED and suicidal ideation, and this association also did not differ across ethnic minority groups.

### Key findings

Consistent with previous US studies,^14–16,34,35^ in hypothesis one, we found a positive association between PED and suicidal ideation among ethnic minority groups in The Netherlands. However, the association in our sample is much weaker than in the previous studies. A possible explanation for this discrepancy might be the difference in age of the study samples, since most previous studies included selective samples, mostly African American adolescents and highly educated young adults,^13,15,16,35–37^ in contrast to our sample, which included a wider age range (18–70) and different multi-ethnic minority groups.^14–16,35^ Since suicide is a leading cause of death among people aged 10–24 years, with the majority of these young people being from an ethnic minority background,^38^ this might indicate higher rates of suicidal ideation in this specific age group.^13,15,16,36^ This specific population is also more susceptible to engaging in suicidal behaviour,^39^ due to a critical phase in which neuronal and self-development takes place.^40^ Moreover, the experience of the adverse consequences of ethnic discrimination contributes negatively towards suicidal ideation.^41^ Together, these findings suggest that the strength of the association between PED and suicidal ideation might differ between age groups.

Contrary to hypothesis two, the present study did not find that the association between PED and suicidal ideation differed across different ethnic minority groups. Previous studies within the same study sample did find stronger associations among other ethnic minority groups concerning the association between PED and other health outcomes.^17,25^ However, previous US research on the influence of ethnicity on the association between PED and suicidal ideation was inconclusive, which could be the result of heterogeneous populations in the selected studies (e.g. different ages and ethnic minority groups).^13,15,36^ Together, these results indicate the complex multifactorial relationship between PED and negative health outcomes in different ethnic minority groups. Future (longitudinal) studies should therefore aim to investigate a more extensive set of factors (for instance religiosity), which might differ between ethnic groups and could explain differences in PED and negative health outcomes.

Regarding our third hypothesis, we found that mastery did not moderate the relationship between PED and suicidal ideation and that this did not differ between the ethnic minority groups. Despite the fact that Slotman et al (2017)^17^ confirmed the moderating effect of mastery on PED and (psychological) well-being and Noh et al (1999)^42^ proposed a buffering effect of mastery on the association between PED and suicidal behaviour, to the best of our knowledge, no previous studies have reported on the moderating effect of mastery in the association between PED and suicidal ideation. Mastery can be seen as a coping resource, and the way individuals tend to cope with PED depends on the type of coping style they use.^42^ Although we did not find a moderating effect of mastery on the studied association, mastery in itself was associated with lower levels of suicidal ideation, indicating that high levels of mastery are associated with lower suicidal ideation but not through buffering the negative effects of PED. This emphasises that mastery is a possible target for reducing suicidal ideation, and future (longitudinal) research should investigate whether interventions aimed at improving mastery could reduce suicidal ideation in ethnic minority groups.

Our sensitivity analyses showed that controlling for depressive symptoms influenced the association between PED and suicidal ideation, as the results attenuated when depressive symptoms were included as a covariate. These findings are in line with previous studies and are not surprising since depressive symptoms are a well-characterised contributing risk factor for suicidal ideation and vice versa.^25,43^ However, the attenuating effect we found was minor, as no major differences in comparison with the main analyses appeared. This suggests that PED is a contributing factor for suicidal ideation, partly independent of depressive symptoms.

### Strengths and limitations

The current study provides new insights into the association between PED and suicidal ideation and the moderating role of ethnicity and mastery specifically among ethnic minority groups in The Netherlands. We included a large sample of different ethnic minority groups, which is representative of The Netherlands. Further, the present study used self-reporting questionnaires, which is a strength, as it provides a better representation of respondents’ perspectives, especially because of the topic's sensitivity (PED, suicidal ideation).

However, it is not without limitations. First, the current study has a cross-sectional design; consequently, no causal inferences can be drawn from our results. Psychological characteristics (e.g. suicidal ideation, mastery and depressive symptoms) can also have a negative impact on perceived discrimination. Future longitudinal research is warranted to address the directionality of the relationships between the investigated variables. Second, we used the suicide item of a scale primarily designed to measure depression severity to capture suicidal ideation.^44^ However, our approach is in line with most research and the use of a single item proved to be a valid approach to assess suicidal ideation.^27^ Additionally, a limitation is that this item covers not only suicidal ideation but also thought of other kinds of self-harm. Despite the fact that the same questionnaire was used to assess depressive symptoms (items 1–8) and suicidal ideation (item 9), the use of the PHQ-9 without item 9 has been proven to reflect depressive symptoms adequately.^45^ Moreover, we did not include Structured Clinical Interview for DSM-5 (SCID) score (or DSM-IV/DSM-5 diagnosis) as a variable in the analysis to clinically assess depressive symptoms. Future studies should measure suicidal ideation using validated instruments specifically developed for the purpose, such as the Colombia Suicide Severity Rating Scale.^46^ Furthermore, it is not known whether the interpretation of questions regarding suicidal ideation and mastery are similar across different ethnic groups and this might have influenced the results.^47^ Last, this study included the feeling of being discriminated against, although the person might not be discriminated according to the law. In this way, potential discrimination is considered.

### Implications

Our finding of a small positive association between PED and suicidal ideation that did not significantly vary between ethnic minority groups. This further supports current evidence of a negative role of PED in determining (mental) health. Moreover, this emphasises that, apart from discrimination as a social problem, PED can have mental health consequences, including suicidal ideation. Therefore, our findings suggest the importance of studying the effect of reducing ethnic discrimination to prevent suicidal ideation. Although the current study did not find evidence for mastery as a potential moderator of the association between PED and suicidal ideation, high mastery was associated with lower suicidal ideation, emphasising the potential role of mastery as a target for reducing suicidal ideation. Besides ethnic discrimination being part of a larger, societal problem which should be targeted, future (longitudinal) research is therefore needed to investigate whether improving mastery in ethnic minority groups could reduce suicidal ideation.

## Data Availability

The data that support the findings of this study are available on request from the HELIUS research cohort, although restrictions apply to the availability of these data, which were used under license for the current study and are not publicly available. Ms Henrike Galenkamp is the Data Collection Coordinator of HELIUS and may be contacted with further questions (h.galenkamp@amsterdamumc.nl).
